# Cotton leaf disease detection model focusing on small targets and comprehensive feature extraction

**DOI:** 10.1038/s41598-025-24898-5

**Published:** 2025-11-20

**Authors:** Halidanmu Abudukelimu, Gengrong Zhang, Abudukelimu Abulizi, Junxiang Ye, Mayilamu Musideke, Yaqing Shi, Gulimire Awudan

**Affiliations:** College of Information Management, Xinjiang University of Finance and Economics, Urumqi, 830012 China

**Keywords:** Plant physiology, Computer science, Image processing

## Abstract

Cotton, as a globally important economic crop, requires early and accurate disease detection to ensure stable yield and promote sustainable development. However, due to the small size of certain leaf lesions, traditional detection methods often suffer from missed or false detections. To address this issue, we propose an improved YOLOv8-based model, CM-YOLO, aimed at enhancing the detection performance for small cotton leaf disease targets. Specifically, the SS2D module from VMamba is introduced into the backbone network to achieve comprehensive feature extraction through multi-directional scanning. Furthermore, the MSDA module is embedded prior to the SPPF module to reduce performance degradation caused by redundant computations and to enhance the model’s focus on critical small targets. Finally, the original bounding box loss function is replaced with DIoU, enabling precise localization of small targets by optimizing anchor center point distances and accelerating model convergence. Experimental results demonstrate that CM-YOLO achieves superior performance in cotton leaf disease detection, with an mAP50 of 0.933 and a recall of 0.891. Compared with state-of-the-art methods, YOLOv8n and YOLOv11n achieve mAP50 values of 0.874 and 0.930, respectively, both lower than CM-YOLO, thereby validating the effectiveness of the proposed method. Additionally, generalization experiments indicate that the model maintains high detection accuracy and robustness across different plant datasets, highlighting its strong applicability in complex scenarios and providing a valuable reference for intelligent agricultural disease detection research.

## Introduction

Cotton, one of the most important economic crops globally, is widely used in the textile industry as well as in oil production. As a strategic sector, it significantly influences the national economy and people’s livelihoods, impacting global agricultural markets and downstream textile trade patterns^1,2^. Cotton is used in many areas. For example, in healthcare, absorbent cotton is essential for managing bleeding^3^. Consequently, growing cotton significantly influences farmers’ incomes and supports the nation’s economic growth.

Cotton leaves, as a crucial organ in the growth and development of the plant, are vital for photosynthesis and have a direct impact on both the yield and quality of cotton. By monitoring the condition of cotton leaves, growers can manage cotton growth more precisely^4^. However, the state of cotton leaves is susceptible to various biotic and abiotic stresses, such as pests, drought, and salinity, which can lead to significant declines in cotton yield and affect fiber quality, thereby posing serious threats to the stability of cotton production and supply chains. Thus, timely and accurate detection of pest damage on cotton leaves is crucial for maintaining stable and sustainable cotton production.

Traditional methods for cotton leaf detection primarily include visual inspection, manual sampling, pest and disease identification charts^5^, environmental monitoring^6^, and pesticide traps^7^. Visual inspection relies on the observer’s experience to identify signs of pests and diseases by observing the color, shape, and surface condition of cotton leaves. While simple, it may lead to missed detections^8^. Manual sampling involves randomly collecting samples from cotton fields at regular intervals to check for pest damage or disease spots. Although it provides specific information, it is time-consuming and labor-intensive. Pest and disease identification charts improve accuracy by comparing with known images of pests and diseases, but they require a high level of expertise. Environmental monitoring records factors such as climate and soil moisture to predict the risk of pest occurrence, aiding in early prevention, but it cannot directly detect current pest conditions. Pesticide traps assess pest population density and activity by capturing specific insects and periodically checking the number caught. Although these traditional methods are somewhat effective, they are inefficient and struggle to meet the modern agricultural demand for rapid and accurate detection. Therefore, an efficient method for detecting cotton leaves is urgently needed to enhance production efficiency.

Advancements in computer technology and modern agricultural science have resulted in an increased reliance on object detection models for detecting cotton leaves. Object detection has progressed through two primary stages: early object detection and deep learning-based object detection. Traditional methods are relatively simple, relying on manually designed features, which can be a labor-intensive and time-consuming process. Furthermore, these traditional methods exhibit limited generalization capabilities and struggle in complex scenarios. In contrast, deep learning models can automatically learn effective features from large-scale agricultural data, enabling high-precision recognition and monitoring of crops, diseases, and pests. These models have been widely applied in agricultural image analysis, substantially improving the efficiency and accuracy of agricultural management^9,10^. In this context, deep learning-based object detection models achieve precise localization and identification of various targets by automatically extracting image features. Classic object detection models, such as YOLO (You Only Look Once)^11^, not only provide precise recognition in complex environments but also enhance detection accuracy and speed through various model improvements, effectively meeting the requirements for real-time monitoring.

**Fig. 1:**
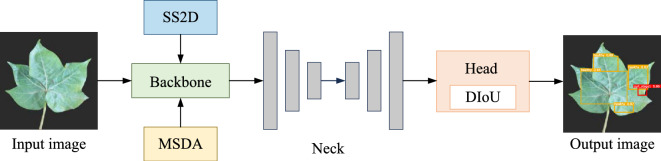
CM-YOLO Model Architecture.

However, due to the high similarity in features such as morphology and color among different conditions of cotton leaves, the YOLO model faces significant challenges in classification. To address this problem, Noon et al.^12^ enhanced the YOLO model^13^ to improve the convergence speed of the network. However, the features of some lesions on cotton leaves occupy a relatively small area in the image, which makes it difficult for the YOLO model to capture the subtle features of these lesions. This limitation leads to low recognition precision and classification accuracy when detecting cotton leaf diseases. In this context, Zhang et al.^14^ developed an improved YOLOv8 model, incorporating the GSConv module^15^ to detect leaves damaged by cotton aphids, enhancing the model’s multi-scale feature fusion capabilities while reducing its complexity, but the increase in detection accuracy was not significant.As shown in Fig. 1, we proposed a robust detection method for assessing cotton leaf condition using an enhanced YOLOv8 model to address these challenges. The main contributions of this work are as follows:

- We introduce the SS2D module from the VMamba model into the backbone of YOLOv8 to enhance long-range dependency capabilities, thereby ensuring that the model achieves a global receptive field at linear complexity and comprehensively extracts image features through multi-directional scanning.
- We integrate the MSDA module before the SPPF module at the end of the YOLOv8 backbone, reducing redundant computations by simulating the interactions between small-scale sparse image patches.
- We incorporate the original loss function of YOLOv8 with DIoU, streamlining the loss calculation and allowing the detection head to focus on anchor box overlap and central position distance, which ultimately improves model accuracy.

The structure of this paper is organized as follows: Section “Related work” presents a brief overview of related work in both object detection and cotton leaf detection. Section "CM-YOLO" elaborates on the proposed CM-YOLO model, detailing its overall architecture and implementation. In Section “Experiment”, we introduce the dataset, experimental setup, and evaluation metrics. Section "Experiment results and analysis" provides a comprehensive description of the experiments conducted in this study, while Section “Conclusion” concludes with a summary of the findings.

## Related work

### Object detection

With the advancement of technology, deep learning models have found widespread applications in various domains, such as ship detection, medical image diagnosis, and agricultural monitoring^16–18^. As a representative task, object detection not only requires models to accurately localize and identify objects within images but also demands efficient capability to process massive amounts of high-resolution images in real time. Currently, object detection is primarily divided into two main approaches: two-stage detection and one-stage detection.

Two-stage detection employs an advanced strategy by breaking the target detection task into two sequential steps. First, potential target areas are identified using the Region Proposal Network (RPN). Next, in the second stage, these candidate regions undergo precise classification and bounding box regression. It has higher detection accuracy, especially in complex background and small object detection. R-CNN^19^ is a groundbreaking algorithm in two-stage detection. It utilizes the selective search method to generate candidate boxes, which are resized to a fixed dimension before being fed into a convolutional neural network (CNN)^20^ for feature extraction. Spatial Pyramid Pooling Networks (SPP-Net)^21^ is an improved CNN architecture that can process input images of any size, overcome the limitation of traditional CNN on fixed input size, and can effectively capture spatial information at different scales. It enhances both target detection and image classification performance while speeding up the training process, establishing a crucial foundation for future research in target detection. After that, Fast R-CNN^22^ and Faster R-CNN^23^ are important advancements in the field of deep learning target detection. Fast R-CNN inputs the entire image into CNN to generate a feature map, and uses selective search to generate candidate regions, thereby improving detection speed and accuracy. On this basis, Faster R-CNN introduces RPN to achieve end-to-end training, directly generating high-quality candidate regions on the feature map, eliminating the reliance on selective search, and further improving detection efficiency and accuracy. Although the two-stage detection method improves accuracy through RPN, its network structure, slow processing speed and strict hardware requirements lead to poor real-time performance.

To address the speed and efficiency constraints of traditional two-stage object detection methods, one-stage models have been developed. One-stage detection treats object detection as a regression problem, directly predicting the object category and location in a single inference. This approach enhances speed, achieves high computational efficiency, and is well-suited for real-time applications. The YOLO^11^ model, which directly optimizes detection performance end-to-end through a single network, was proposed in 2016. This approach frames object detection as a regression task that focuses on spatially distinct bounding boxes and their associated class probabilities. It directly predicts these bounding boxes and class probabilities from the entire image in a single pass. It has a fast speed and strong generalization ability, which greatly improves the efficiency of the object detection model. Based on the one-stage idea of YOLO and the anchor box method of Faster RCNN^23^, SSD^24^ has good real-time performance, but the detection accuracy is low. Based on the encoder-decoder architecture of the global loss and Transformer model, DETR (DEtection TRansformer)^25^ can infer the relationship between the target and the global image context by giving a fixed set of learning object queries, and directly output the final prediction set in parallel. It offers impressive processing speed, making it more suitable for deployment on mobile devices.

So far, the YOLO series^26–31^ has undergone multiple iterations and improvements, and has performed well in the agricultural field, but it still has problems with low precision and low classification accuracy. Based on this, we use YOLOv8n as the basic model, combines the SS2D module and the MSDA attention mechanism, and improves the model performance of cotton leaf detection by replacing the loss function.

### Cotton leaf detection

As an important cash crop, cotton’s yield and quality directly affect the country’s economic stability. However, cotton is susceptible to a variety of diseases during its growth, leading to leaf damage and significantly reducing yield and fiber quality. As the scale of global cotton cultivation continues to expand, timely and accurate detection and identification of these diseases is crucial for cotton production.

Traditional cotton leaf detection methods rely on manual observation and empirical judgment, which is not only time-consuming and easily affected by subjective factors, but also often fails to detect diseases in a timely manner. In addition, with climate change and the complexity of the agricultural environment, the types and occurrence patterns of diseases are also constantly changing, which puts higher demands on traditional detection methods.

Therefore, the development of efficient and automated cotton leaf disease detection technology has become an urgent need for modern agricultural management. Using advanced image processing and deep learning technology, rapid and accurate detection of cotton leaf diseases can be achieved, helping cotton growers take timely measures to reduce losses caused by diseases.

In early work, many researchers have devoted themselves to using image processing technology to study cotton leaf detection related issues. Revathi et al.^32^ used image RGB feature ranging technology to identify diseases and identified disease points by identifying edge features. Ma et al.^33^ used high-resolution RGB images from drones and different feature screening methods to monitor cotton leaf shedding and cotton boll opening. He et al.^34^ compared cotton leaves with different levels of mite infestation and used the mean and variance of color features to automatically monitor mite infestation, achieving good results. However, image processing technology alone is not only inefficient, but also has the problem of low accuracy.

With the development of computer technology and agricultural informatization, domestic and foreign scholars have applied machine learning (ML) technology to conduct certain research on automatic identification of crop pests and diseases. Li et al.^35^ used Fourier transform infrared (FTIR) spectroscopy to detect yellow wilt infection in cotton leaves. By comparing the classification models constructed by different machine learning algorithms, they discovered that the support vector machine (SVM) model^36^ showed the highest prediction accuracy. Gao et al.^37^ combined the extreme learning machine^38^ and Sparrow search algorithm optimization model, which demonstrated the robustness, adaptability and generalization of predicting cotton leaf water potential based on RGB images combined with data in different geographical environments. Sain et al.^39^ compared the performance of multiple linear regression (MLR)^40^, bootstrap forest (BSF)^41^, and other ML models^42,43^ in predicting leaf curl in field cotton. The results showed that the BSF model performed best among these ML models. Harish et al.^44^ applied the CatBoost algorithm^45^ to the dataset for training, thereby creating a highly accurate model file and generating the optimal discriminant model, providing data support for early diagnosis of cotton leaves.

Nowadays, cotton leaf detection based on deep learning technology has achieved higher performance. Feng et al.^46^ developed an efficient, high-throughput, automated cotton whitefly rapid identification and quantification tool based on the YOLOv8 model. By replacing the C2f module of the YOLOv8 model with Swin-Transformer^47^ and introducing the P2 structure in the head, accurate recognition of small targets can be achieved. Ahmed et al.^48^ developed a high-performance model for detecting cotton diseases from leaves to reduce yield losses caused by cotton diseases and help farmers easily detect diseases before they spread to crops. By comparing the SVM-VGG16^49^ hybrid model and the MobileNet model^50^, the latter achieved an accuracy of 99%. Heba et al.^51^ used the ResNet50 model to extract features from cotton leaf images and combined it with the Gray Wolf optimization algorithm to improve the model performance by 78.57%. To solve the high cost problem, Zhang et al.^52^ introduced the focal loss function based on the ResNet50^53^ network model, embedded the attention mechanism modules in different network layers, and added Dropout regularization to construct an improved ResNet50 model, which reduced the computational cost and improved the accuracy.

As the data set increases, the real-time problem of the model is difficult to solve. To deal with this challenge, Jayanthy et al.^54^ combined with the lightweight MobileNetV2^55^ to achieve higher accuracy and verification speed in cotton leaf detection work. Wang et al.^56^ introduced the ResNet-50 structure into the improved Transformer-DETR^25^ model, which showed significant advantages in balancing lightweight and accuracy. Zhang et al.^57^ introduced the residual module in Swin Transformer^47^ to improve the model detection accuracy while shortening the detection time.

In summary, deep learning technology has advanced greatly in cotton leaf detection, with the use of different models consistently enhancing detection performance and efficiency. Nonetheless, low model accuracy remains a challenge that must be addressed. To enhance the efficiency and accuracy of cotton leaf disease detection, this study introduces an improved model designed to optimize the detection process and fulfill practical application requirements.

## CM-YOLO

This section briefly describes the base model YOLOv8 and the proposed CM-YOLO for cotton leaf detection. Subsequently, we explore the details of the method, including the SS2D module, MSDA module, and loss function, which will be elaborated in the following subsections.

**Fig. 2:**
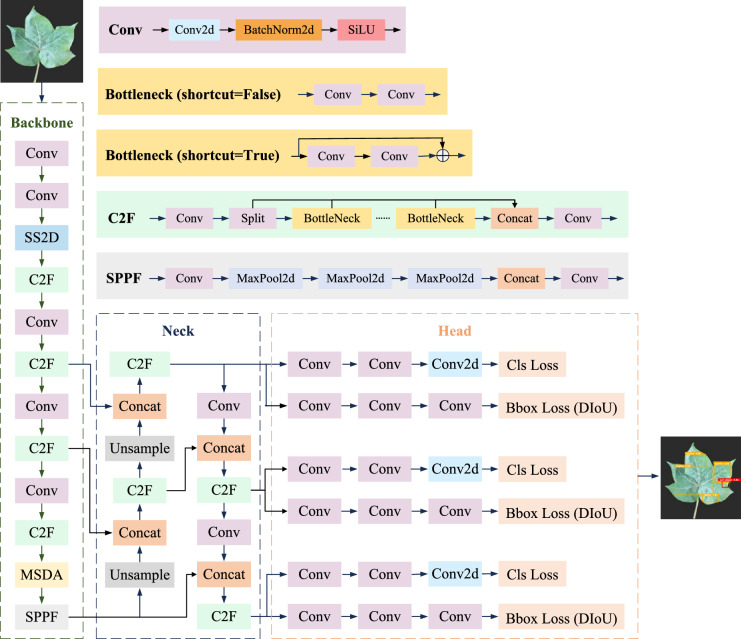
CM-YOLO network structure diagram.

As shown in Fig. 2, the model’s architecture is organized into three key modules: Backbone, Neck, and Head. The Backbone module handles the initial feature extraction, incorporating multiple convolutional layers (Conv) along with the SS2D^58^, C2F, and MSDA modules^59^, before ultimately connecting to the SPPF module. The SS2D module strengthens the model’s ability to capture long-range dependencies, while the MSDA module enhances robustness in detecting targets of varying scales and shapes, contributing to improved detection accuracy. The Neck module focuses on feature fusion, using multiple upsampling (Upsample) and concatenation (Concat) operations to integrate features from different levels, thus enriching feature representation. Finally, the Head module performs target classification (Cls Loss) and bounding box regression (BBox Loss), with the standard loss function replaced by the DIoU loss function^60^ to achieve more accurate bounding box localization.

### SS2D

In the cotton leaf detection task, two main challenges arise: the small size of the detection targets and the visual similarity between different states of cotton leaves. These factors affect the model’s ability to accurately and efficiently differentiate and identify cotton leaf diseases. Therefore, the model must simultaneously focus on the integration of global semantic information and local detailed features, achieving comprehensive contextual modeling while performing refined feature extraction and mining, thereby enhancing its ability to capture subtle variations and discriminative characteristics within images^61,62^. Recently, researchers have successfully introduced the Mamba model into the field of computer vision, achieving notable success in image processing^63–65^, providing a solution to the above problems.

The Mamba model is based on the concept of the state space model (SSM)^66^ and aims to improve the efficiency of traditional architectures. Unlike the Transformer model^67^, which relies on the attention mechanism, the Mamba model dynamically adjusts the information to be retained or discarded based on the context, achieving linear time reasoning and parallel training for long context tasks, thereby processing long sequences more effectively^58^. Compared to the Transformer, the Mamba model offers the advantage of linear time scalability, making it faster and more memory-efficient when handling long sequences.

1 $$\begin{aligned} h'(t) = Ah(t)+Bx(t) \end{aligned}$$

2 $$\begin{aligned} y(t) = Ch(t)+Dx(t) \end{aligned}$$

where $x(t) \in \mathbb {R}$ is the input sequence, *y*(*t*) is the output sequence, $h(t) \in \mathbb {R}^N$ is the implicit potential intermediate state, $A \in \mathbb {R}^{N \times N}$ represents the state transfer matrix, which controls how the hidden state evolves over time, $B \in \mathbb {R}^N$ represents the weight matrix of the input space relative to the hidden state, $C \in \mathbb {R}^N$ is the observation matrix, which maps the hidden intermediate state to the output, and $D \in \mathbb {R}^1$ is the jump connection. This design not only realizes the connection relationship between input and output, but also encapsulates the temporal dynamics.

The 2D-selective-scan (SS2D) module of VMamba (Vision Mamba) is a key component that influences the number of parameters. The SS2D module encompasses scan expansion operations, feature extraction using the Selective Scan Space State Sequential Model (S6)^68^, and scan merging operations. S6 serves as the core operator that ensures the dynamic adjustment of weights within the mechanism.

**Fig. 3:**
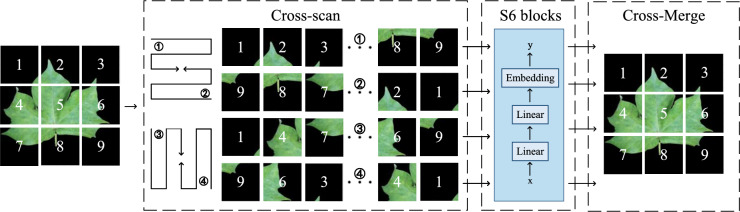
Image processing flow of SS2D module.

To effectively extract cotton leaf features from images, we incorporate the SS2D module into YOLOv8’s feature extraction component. As illustrated in Fig. 3, since images lack a natural order like text sequences, the scan expansion operation is employed to divide the input image into a series of sub-images. Each sub-image represents a specific direction and is scanned diagonally in four symmetrical directions (top-down, bottom-up, left to right, and right to left). This approach provides thorough coverage of the input image while building a rich, multi-dimensional information base for later feature extraction. It enhances the efficiency and comprehensiveness of multi-dimensional feature capture and flattens the 2D features into 1D vectors, effectively reducing quadratic complexity to linear. The four resulting 1D vectors are then input into the S6 block for feature extraction. Unlike the attention mechanism in ViT (Vision Transformer), Mamba’s S6 block introduces a selection mechanism on top of S4^69^, enabling the model to identify and retain essential information while discarding irrelevant data. The S6 block interacts each element in the 1D vector with the information scanned prior to it, capturing different features and further reducing quadratic complexity to linear. Finally, these sub-images are merged through scan merging to produce an output image of the same size as the input image.

### MSDA

In image processing tasks, ViT divides the image into multiple small blocks based on the Transformer architecture, and models these small blocks to extract features. Its global dependency modeling considers the relationship between all small patches in the image when calculating, allowing the model to capture long-distance contextual information, but it will lead to a significant increase in computational complexity, making the computational cost too high, and thus wasting computational resources. In cotton leaf detection, real-time performance and efficiency are more important. The computational expense of ViT can lead to prolonged model response times, rendering it unsuitable for real-time detection tasks. Therefore, this article uses the Multi-Scale Dilated Attention (MSDA) mechanism^59^ to obtain long-distance contextual information at different scales, reduce redundant calculations, and thereby improve model performance.

Since the patches in the partial attention matrix are sparsely distributed around the query value, global attention often introduces a lot of redundancy. The sliding window dilated attention (SWDA) mechanism addresses this issue by selectively calculating self-attention within a sliding window, which sparsely targets key and value features. This method can efficiently model long-distance context.

3 $$\begin{aligned} X = SWDA(Q, K, V, r) \end{aligned}$$

Let *Q*, *K*, and *V* denote query, key, and value, respectively. The SWDA module computes self-attention over a sliding window of size $w \times w$ with a specified dilation rate *r*. The output vector $x_{ij}$ of *x* is:

4 $$\begin{aligned} x_{ij} = Attention(q_{ij},K_r,V_r)=Softmax(\frac{q_{ij}K_r^T}{\sqrt{d_k}})V_r \end{aligned}$$

Here, *H* and *W* represent the height and width of the cotton leaf image, respectively. $K_r$ and $V_r$ represent the key and value.

Building on the concept of SWDA, the MSDA module assigns varying dilation rates to different heads to better leverage the information. This approach captures semantic information at multiple scales, facilitating multi-scale learning.

**Fig. 4:**
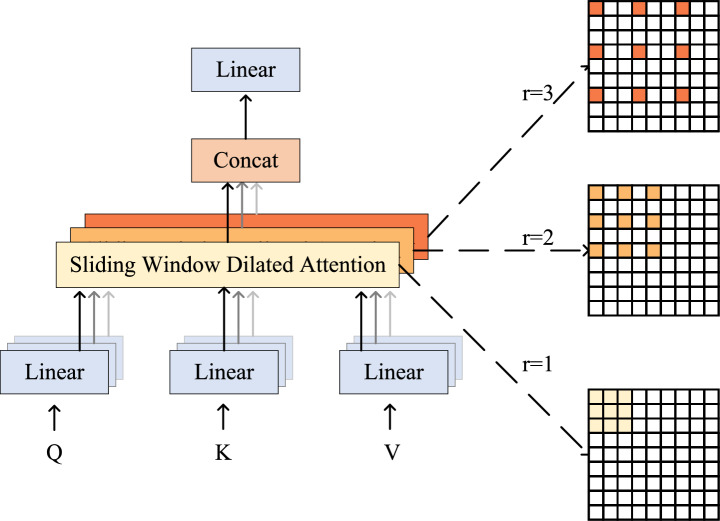
Framework of the MSDA Module^59^.

As shown in the Fig. 4, the input image *X* generates queries, keys, and values through linear projection. Then the channels of the graph are divided into *n* different heads and calculated using different dilation rates. Finally, the output vectors are merged and input to the next layer.

5 $$\begin{aligned} h_i = SWDA(Q_i, K_i, V_i, r_i), 1 \le i \le n \end{aligned}$$

6 $$\begin{aligned} X = Linear(Concat[h_1,...h_n]) \end{aligned}$$

### DIoU

In deep learning, loss functions are typically employed to assess the disparity between a model’s predictions and the actual results. An appropriate loss function can greatly improve the precision of object detection. Common bounding box loss functions in object detection include CIoU^70^, GIoU^71^, SIoU^72^, DIoU^60^, and EIoU^73^. In the loss functions mentioned above, the intersection over union (IoU)^74^ assesses model accuracy by measuring the overlap ratio between the predicted box and the ground truth box.

7 $$\begin{aligned} IoU = \frac{|A \cap B|}{|A \cup B|} \end{aligned}$$

The baseline model uses CIoU as the loss function. CIoU builds on the IoU concept by adding two penalty terms to address the distance between central positions and the variations in aspect ratios of the bounding boxes.

8 $$\begin{aligned} CIoU = IoU-(\frac{\rho ^2(b,b^{gt})}{c^2}+\alpha v) \end{aligned}$$

9 $$\begin{aligned} c = \sqrt{(x_{max}-x_{min})^2+(y_{max}-y_{min})^2} \end{aligned}$$

10 $$\begin{aligned} \alpha = \frac{v}{(1-IoU)+v} \end{aligned}$$

where $\alpha$ is the weight function, *v* measures the consistency of aspect ratio, *w* and *h* are the width and height of the predicted box, and $w_{gt}$ and $h_{gt}$ are the width and height of the real box.

**Fig. 5:**
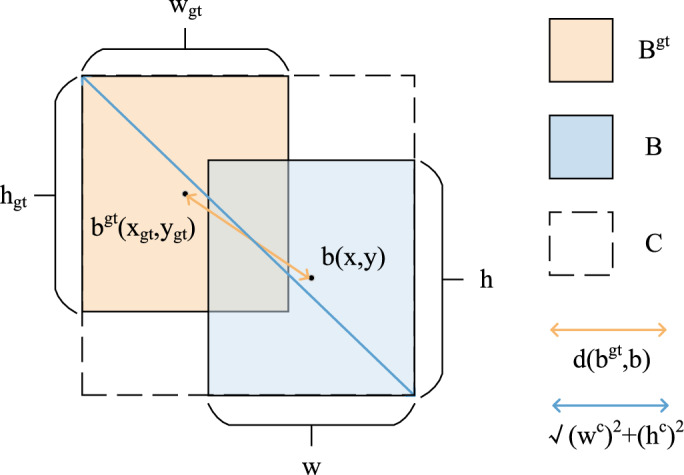
Computational Diagram of the DIoU Loss Function.

However, in the cotton leaf detection task, the targets may be small and have various shapes. Although CIoU accounts for the aspect ratio, in scenarios with small targets, the aspect ratio may have a greater impact than position. As illustrated in Fig. 5, DIoU takes into account the distance, overlap ratio, and scale between the predicted and ground truth boxes. By minimizing the distance between the central positions of these boxes, the regression of the predicted box stabilizes and converges more rapidly to the correct position, enhancing the positioning accuracy of the detection box and making it more effective for cotton leaf detection.

11 $$\begin{aligned} DIoU = IoU- \frac{\rho ^2(b,b_{gt})}{c^2}=IoU- \frac{d^2}{c^2} \end{aligned}$$

Here, *d* represents the distance between the central positions of the two anchor boxes, and *c* denotes the diagonal length of the minimum bounding box enclosing both anchor boxes.

## Experiment

This section begins by detailing the dataset and describing the specific symptoms of cotton leaf disease. It then introduces the dataset enhancement methods and model evaluation indicators, concluding with a description of the experimental environment used in this work.

### Dataset

#### Dataset preparation

**Fig. 6:**
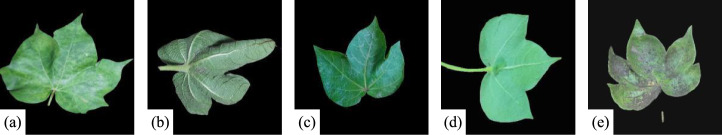
Five states of cotton leaves: (**a**) curl_stage1; (**b**) curl_stage2; (**c**) healthy; (**d**) leaf_enation; (**e**) sooty.

Currently, studies on cotton leaf disease detection primarily focus on identifying different severity stages. However, in actual production, leaf disease often affects the overall health of the whole leaf, varying in morphology and size. Therefore, the data in this study is a cotton leaf disease dataset available on the Roboflow platform^75^, consisting of 1,183 filtered cotton leaf images captured using smartphone cameras in both controlled and real-world environments. This dataset encompasses a variety of scenarios, including the presence of multiple stresses co-occurring on a single leaf and the progression of disease severity, with various diseases and severity levels coexisting on one leaf. In addition, the dataset includes five cotton leaf conditions, as shown in Fig. 6, which help to visually present the lesion characteristics, namely curl_stage1, curl_stage2, healthy, leaf_enation, and sooty.

Leaf curl is a viral disease that causes cotton leaves to curl and exhibit abnormal colors after infection, resulting in poor growth and reduced yields. leaf_enation, on the other hand, is a plant disease caused by fungi that primarily affects the leaves, flower buds, young leaves, and young fruits of plants. Infected areas swell and thicken, often accompanied by a white powder or discoloration on the surface. There are many types of sooty mold, and a single plant can be infected by multiple pathogens. Symptoms include the formation of small black mold spots on leaves and branches, which can expand and merge, ultimately covering the entire leaf surface and tender branches with layers of black mold, potentially leading to plant death.

#### Data enhancement

To increase the diversity and richness of the training sample images so as to enhance the quality as well as effectiveness of the model training. The data augmentation was performed on the original cotton leaf disease dataset, and the augmentation methods include flipping, rotating, adjusting exposure, saturation, blur, and brightness, and adding noise.The dataset was eventually expanded to 5,533 images.

### Evaluation metrics

To comprehensively evaluate the model’s performance in cotton leaf detection, we employ five metrics. These metrics offer a comprehensive perspective on the model’s effectiveness regarding precision, recall, speed, and complexity.

**Precision (P)** measures the ratio of correctly predicted positive samples to the total number of samples predicted as positive.

12 $$\begin{aligned} P = \frac{TP}{TP+FP} \end{aligned}$$

**Recall (R)** measures the proportion of correctly predicted positive samples relative to the total number of actual positive samples.

13 $$\begin{aligned} R = \frac{TP}{TP+FN} \end{aligned}$$

**Mean Average Precision (mAP)** represents the average of the mean average precisions across multiple classes.

14 $$\begin{aligned} AP = \int _{0}^{1} P(R) \, dR \end{aligned}$$

15 $$\begin{aligned} mAP = \frac{\sum _{i=1}^{n} AP_i}{n} \end{aligned}$$

In addition, the model’s size, number of floating-point operations, and frames per second (FPS) were also evaluated.

### Implementation details

All experiments in this study were conducted in a consistent environment, utilizing an NVIDIA 4090D GPU with 24GB of memory, CUDA 12.1, and a Linux operating system, with PyTorch 2.3.0 as the deep learning framework and Python 3.8 as the programming language. During model training, the input size was set to 640×640, with a batch size of 32 over 200 epochs. Additionally, we used SGD as the optimizer, with an initial learning rate of 0.01.

## Experiment results and analysis

### Performance comparison with different detection models

To comprehensively evaluate the performance of CM-YOLO, we compared it with several mainstream object detection models, including Faster R-CNN, RetinaNet, DETR, the YOLO series, RT-DETR, Gold-YOLO, and MambaYOLO. Performance metrics included P, R, mAP@50, mAP@50–95, number of parameters (Params), GFLOPs, and inference speed (FPS). This evaluation not only demonstrates the high performance of the model but also highlights its efficiency and lightweight design, allowing readers to gain a comprehensive and intuitive understanding of the model’s advantages across multiple dimensions and providing references for practical applications.

The experimental results, as shown in Table 1, indicate that CM-YOLO achieves excellent overall detection performance. Specifically, its precision reaches 0.902, slightly lower than YOLOv3 (0.931) but significantly higher than YOLOv8n, indicating that CM-YOLO effectively reduces false positives while maintaining high accuracy. This suggests that the model can better distinguish diseased regions from healthy leaves, minimizing misclassification. In terms of recall, CM-YOLO achieves 0.891, the highest among all compared models, representing an improvement of approximately 6.8% over the baseline YOLOv8n and the MambaYOLO model, which also incorporates Mamba-based improvements. This demonstrates its remarkable capability in detecting small and subtle disease spots, ensuring comprehensive coverage of diseased areas. For mAP@50, CM-YOLO attains 0.933, the highest among all models, reflecting its outstanding ability to detect diverse forms of cotton leaf diseases under a low IoU threshold. Under the stricter mAP@50–95 metric, CM-YOLO achieves 0.663, slightly lower than YOLOv3 and YOLOv11n but still higher than YOLOv8n and YOLOv10n, indicating that it remains highly competitive under high IoU conditions and can accurately capture disease spots of varying sizes and complex shapes. In contrast, DETR performs the worst, primarily due to its end-to-end Transformer architecture, which has limited capability in capturing fine-grained local features for small and densely distributed targets. Its global self-attention mechanism is better suited for large-scale objects, making it prone to missed or incorrect detections for the small and closely spaced disease spots commonly seen in cotton leaves, thereby showing clear limitations in fine-grained disease detection tasks.

**Table 1:** Comparison of cotton leaf detection results by different models.

Method	P	R	mAP50	mAP50-95	Params	GFLOPs	FPS
Faster RCNN^23^	0.719	0.727	0.788	-	-	-	18
RetinaNet^76^	0.860	0.797	0.866	-	-	-	241
DETR^25^	0.570	0.456	0.701	0.472	-	-	8
YOLOv3^26^	**0.931**	0.790	0.872	**0.772**	103668095	282.2	108
YOLOv3-tiny^77^	0.863	0.789	0.843	0.661	12130234	18.9	833
YOLOv5n	0.850	0.819	0.875	0.572	2503919	7.1	1111
YOLOv6n^27^	0.811	0.728	0.789	0.498	4234239	11.8	1110
YOLOv8n	0.863	0.823	0.874	0.598	3006623	8.1	909
YOLOv9t^29^	0.889	0.828	0.892	0.613	**1971759**	7.6	1000
YOLOv10n^30^	0.849	0.824	0.865	0.608	2696366	8.2	1667
YOLOv11n	0.912	0.885	0.930	0.696	2583127	6.2	1111
RT-DETR^78^	0.873	0.830	0.863	0.634	31994015	103.5	86
Gold-YOLO^31^	-	-	0.882	0.588	-	12.1	140
Mamba YOLO^65^	0.890	0.823	0.882	0.652	5984983	13.6	156
CM-YOLO	0.902	**0.891**	**0.933**	0.663	3290527	8.7	417

Moreover, CM-YOLO demonstrates significant advantages in model lightweighting. Its parameter count is only slightly higher than YOLOv11n but substantially lower than YOLOv3. In terms of computational cost, CM-YOLO has 8.7 GFLOPs, far lower than YOLOv3 and MambaYOLO, indicating that the model achieves high efficiency while maintaining strong performance, making it suitable for deployment in resource-constrained environments.

Regarding inference speed, there are notable differences among models. YOLOv5n and YOLOv11n achieve 1111 FPS and 1667 FPS, respectively, primarily due to reduced parameters and simplified network architectures. However, YOLOv5n shows significant declines in precision and recall, limiting its ability to accurately capture fine-grained cotton leaf disease features. In contrast, YOLOv3 exhibits strong precision but an inference speed of only 108 FPS, which is insufficient for resource-limited real-world scenarios. CM-YOLO achieves a balanced trade-off between accuracy and speed, reaching 417 FPS while maintaining high model performance. This advantage is attributed to the lightweight design combined with SS2D and MSDA modules, which optimize feature extraction and small-target detection, enabling the model to capture subtle disease spots effectively while minimizing computational overhead.

In summary, CM-YOLO excels in detection accuracy, recall, mAP metrics, model lightweighting, and computational efficiency. Compared with MambaYOLO, which also incorporates the Mamba module, CM-YOLO further optimizes both detection performance and efficiency, achieving a balance between high performance and high efficiency. Therefore, CM-YOLO is not only suitable for high-precision cotton leaf disease detection but also has strong potential for real-time deployment in practical agricultural production environments.

### Comparative analysis of attention mechanisms

To confirm the performance advantages of the MSDA module, we compared eight attention mechanisms added to the YOLOv8 model alongside the SS2D module. The experimental results demonstrate that MSDA strikes an effective balance between computational efficiency and performance, successfully integrating high detection accuracy and recall with minimal computational cost.

As shown in Table 2, the addition of CBAM and GAM attention mechanisms yielded the least favorable results, with mAP values lower than those of other methods. GAM, which has the highest parameter count among all mechanisms, still recorded a lower mAP than the baseline, possibly due to redundant operations introduced by this attention mechanism. In contrast, the inclusion of MSDA and EMA led to improvements in mAP, with MSDA achieving the most significant increase of 2%. Other attention mechanisms, such as CA, ECA, HAM, and SimAM, also resulted in performance gains, though the improvements were comparatively modest. It can be concluded that the suboptimal performance of certain attention mechanisms is primarily due to the introduction of excessive redundant computations or their inability to effectively capture multi-scale information, whereas MSDA efficiently captures features of varying sizes through multi-scale dilated attention, enabling accurate and effective detection of cotton leaf disease regions.

**Table 2:** Training using different attention mechanisms.

Attention Mechanism	P	R	mAP50	mAP50-95	Params	GFLOPs
CA^79^	0.880	0.852	0.898	0.624	3034039	8.5
CBAM^80^	0.879	0.829	0.886	0.626	3093249	8.5
ECA^81^	0.868	0.854	0.893	0.630	**3027362**	8.4
EMA^82^	0.905	0.846	0.913	0.659	3028031	8.5
GAM^83^	0.850	0.816	0.860	0.582	4667039	9.8
HAM^84^	0.873	0.861	0.908	0.628	3027467	8.4
SimAM^85^	0.876	0.828	0.894	0.613	3027359	8.4
MSDA^59^	**0.902**	**0.891**	**0.933**	**0.663**	3290527	8.7

### Evaluation of loss function performance

To evaluate the performance benefits of DIoU, we compared six distinct loss functions. The experimental results indicate that DIoU offers a balanced precision and recall, along with superior detection performance at lower IoU thresholds.

As shown in Table 3, the P value of DIoU is 0.902, only about 0.8% lower than the highest precision achieved by WIoU, yet it remains at a high level among all loss functions. In terms of R, DIoU reaches 0.891, slightly below EIoU’s 0.896 by only 0.5%, and 0.6% higher than WIoU, demonstrating strong target detection capability. For the mAP50 metric, DIoU achieves the highest score of 0.933, surpassing GIoU’s 0.93 by approximately 0.3%, highlighting its excellent detection performance at lower IoU thresholds. Under the stricter mAP50-95 metric, DIoU scores 0.663, slightly below WIoU’s 0.678 with only a 1.5% difference, while still maintaining stable performance.

**Table 3:** Training using different loss functions.

Loss Function	P	R	mAP50	mAP50-95	Params	GFLOPs
CIoU^70^	0.905	0.871	0.920	0.675	3290527	8.7
EIoU^73^	0.865	0.896	0.926	0.675	3290527	8.7
GIoU^71^	0.882	**0.903**	0.930	0.670	3290527	8.7
SIoU^72^	0.903	0.866	0.916	0.662	3290527	8.7
WIoU^86^	**0.910**	0.885	0.925	**0.678**	3290527	8.7
DIoU^60^	0.902	0.891	**0.933**	0.663	3290527	8.7

### Ablation study on the MSDA module

In order to study how different positions and numbers of MSDA modules affect model performance, we compared five different MSDA addition methods. The experimental results suggest that the 1 MSDA on the main trunk is relatively more effective in detecting multi-scale objects.

As illustrated in Table 4, the experiments with different configurations reveal that integrating a MSDA module into the backbone achieves the most substantial improvement. This improvement is likely due to the backbone of the YOLO model, which primarily focuses on feature extraction. Placing the MSDA module at the end of the backbone, right before the SPPF module, decreases the computational burden and greatly improves the model’s performance by enabling effective deep multi-scale feature learning. Conversely, adding an MSDA module at the end of the head results in the least favorable outcomes, which may be attributed to its inability to effectively leverage preceding features, thereby diminishing performance. While the configurations with three and four MSDA modules at the head better capture multi-scale features, their added complexity does not translate into markedly superior performance compared to a single MSDA. The implementation of one MSDA at the beginning of the head exhibits moderate effectiveness.

**Table 4:** Different positions and numbers of MSDA modules.

MSDA Module	P	R	mAP50	mAP50-95	Params	GFLOPs
Head 3 MSDA	0.885	0.854	0.902	0.636	3373215	9.1
Head 4 MSDA	0.894	**0.900**	0.932	**0.679**	3439263	9.3
1 MSDA at the end of the head	0.900	0.835	0.900	0.618	3290527	8.7
1 MSDA at the start of the head	**0.916**	0.863	0.918	0.672	**3093407**	8.7
1 MSDA on the main trunk	0.902	0.891	**0.933**	**0.663**	3290527	8.7

### Ablation study on module components

To comprehensively analyze the impact of each component: YOLOv8n, SS2D, MSDA, and DIoU on model performance, we carried out an ablation experiment on the detection model. The experimental results highlight that integrating YOLOv8n with SS2D, MSDA, and DIoU achieves optimal performance, with each component contributing to improvements in different areas of the model. This configuration achieves a superior balance between high P, R, and efficient computation, demonstrating the efficacy of incorporating all these elements in a unified model.

**Fig. 7:**
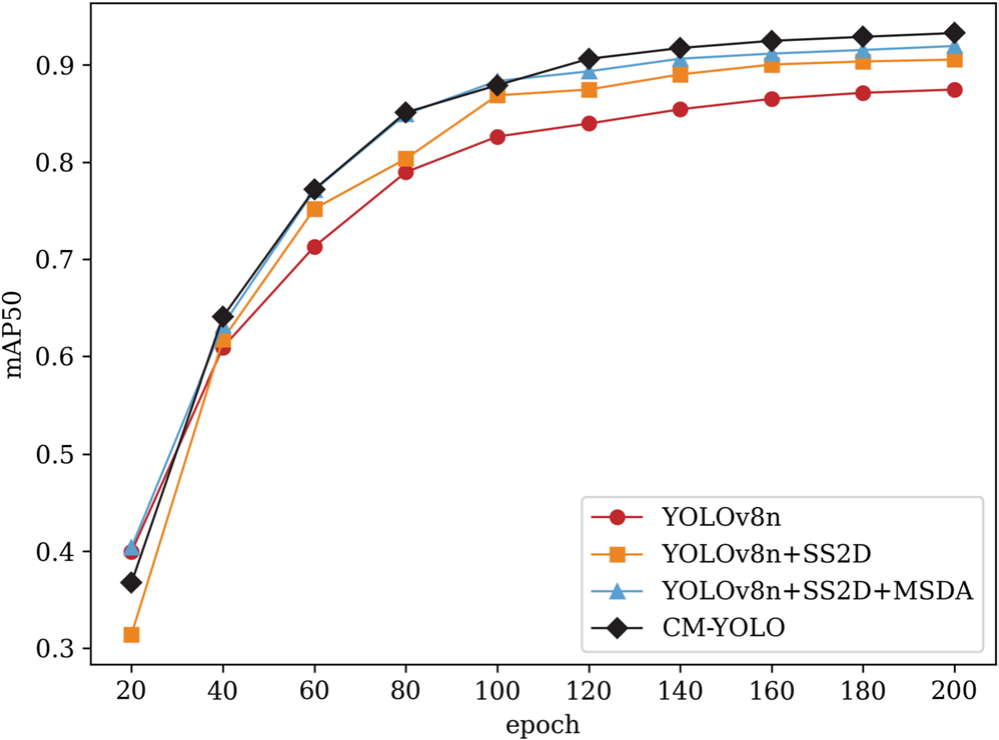
Accuracy curve of the model with different modules superimposed.

As shown in Fig. 7, the configuration with all components achieves the highest performance across multiple metrics, with a P value of 0.902, R of 0.891, mAP50 of 0.933, and mAP50-95 of 0.663. This suggests that incorporating these components enhances the model’s ability to detect and localize objects, leading to a balanced improvement in both precision and recall. Notably, this is achieved without a substantial increase in computational complexity, as evidenced by the relatively low GFLOPs.

As shown in Table 5, breaking down the contributions of each component, it is evident that adding SS2D and MSDA components separately improves the R and mAP scores. For instance, adding SS2D alone to YOLOv8n increases recall to 0.854 and mAP50 to 0.905. When MSDA is added, the model’s P reaches 0.891 and R achieves a peak value of 0.892. This demonstrates that SS2D and MSDA each contribute individually to enhancing certain aspects of the model’s performance, with MSDA providing a notable boost in R.

**Table 5:** Ablation experiment.

YOLOv8n	SS2D	MSDA	DIoU	P	R	mAP50	mAP50-95	Params	GFLOPs
$\checkmark$				0.863	0.823	0.874	0.598	**3006623**	8.1
$\checkmark$	$\checkmark$			0.890	0.854	0.905	0.645	3027359	8.4
$\checkmark$		$\checkmark$		0.891	**0.892**	0.930	0.672	3269791	8.3
$\checkmark$			$\checkmark$	0.877	0.840	0.884	0.600	**3006623**	8.1
$\checkmark$	$\checkmark$	$\checkmark$		0.905	0.871	0.920	**0.675**	3290527	**8.7**
$\checkmark$	$\checkmark$		$\checkmark$	0.866	0.847	0.901	0.618	3027359	8.4
$\checkmark$		$\checkmark$	$\checkmark$	0.891	0.870	0.919	0.667	3269791	8.3
$\checkmark$	$\checkmark$	$\checkmark$	$\checkmark$	**0.902**	0.891	**0.933**	0.663	3290527	**8.7**

Furthermore, the inclusion of DIoU alongside SS2D and MSDA further improves the results, especially in the context of mAP50 and mAP50-95. The combination of YOLOv8n, SS2D, and DIoU yields a respectable mAP50-95 score of 0.675 with minimal increase in parameter count and GFLOPs. This balance between performance and complexity suggests that DIoU, when combined with other modules, contributes effectively to optimizing the trade-off between accuracy and efficiency. The performance improvement stems from the synergistic effect of the three enhanced modules: SS2D enhances feature extraction capabilities, enabling the model to capture subtle textures and local features of cotton leaf diseases; MSDA effectively integrates multi-scale information through dilated attention, significantly improving recall; and DIoU optimizes the matching of the predicted and ground-truth bounding box centers, enhancing localization accuracy and accelerating convergence. The combination of these three modules not only ensures high precision but also improves overall detection performance and efficiency.

### Performance on different types of cotton leaf conditions

To examine the detection outcomes for various cotton leaf states, we compared five specific types. The experimental results reveal that the model performs best in detecting healthy and leaf_enation states, proving its ability to capture distinct features effectively.

As presented in Table 6, the results demonstrate that leaf swelling disease attained the highest performance, achieving an mAP of 99.5% and a recall rate of 100%. This may be due to the limited variety of samples and the lack of additional data for thorough validation. In contrast, the mAP for the first stage of leaf curl disease was the lowest at only 85.8%. This may be due to the visual similarities between the first and second stages of leaf curl disease, which pose challenges for the model in accurately distinguishing between them.

**Table 6:** Comparison of results of different types of cotton leaf states.

Category	P	R	mAP50	mAP50-95
curl_stage1	0.827	0.795	0.858	0.594
curl_stage2	0.911	0.880	0.936	0.698
healthy	0.910	0.880	0.945	**0.749**
leaf_enation	**0.959**	**1.000**	**0.995**	0.597
sooty	0.903	0.900	0.930	0.682

### Statistical significance analysis

To quantitatively assess whether the proposed CM-YOLO model achieves statistically significant improvements over YOLOv8n on key metrics, we conducted paired t-tests. This study employed 5-fold cross-validation, with YOLOv8n and CM-YOLO evaluated on the same test set in each fold, thereby obtaining five paired performance measurements for each model. This approach ensures a fair comparison and provides a stable performance distribution for statistical testing. For each evaluation metric (Precision P, Recall R, mAP50, and mAP50-95), paired t-tests were performed, with the null hypothesis that there is no significant difference in the mean performance between the proposed model and the baseline. The results indicate that CM-YOLO’s improvements on all key metrics are statistically significant (p< 0.05). The detailed results of the 5-fold cross-validation (mean ± standard deviation) and the significance test outcomes are presented in Table 7.

**Table 7:** Statistical Significance Analysis.

Metric	YOLOv8 (Mean ± SD)	CM-YOLO (Mean ± SD)	p-value
P	0.864 ± 0.030	0.906 ± 0.018	0.022
R	0.824 ± 0.050	0.890 ± 0.022	0.027
mAP50	0.875 ± 0.041	0.933 ± 0.020	0.019
mAP50-95	0.596 ± 0.052	0.670 ± 0.025	0.021

Under 5-fold cross-validation, CM-YOLO outperformed the baseline model YOLOv8n across all key metrics. Paired t-test results indicated that these improvements were statistically significant (p< 0.05), demonstrating that the proposed CM-YOLO model can reliably and significantly enhance performance in cotton leaf disease detection tasks.

### Generalization experiments

In practical agricultural production, leaf morphology, lesion characteristics, and color variations are influenced by multiple factors, including crop variety, growth environment, and disease severity, resulting in high diversity. Therefore, evaluating model performance on a single dataset is insufficient to comprehensively assess its adaptability and stability across different environments and disease scenarios^87^. To further validate the generalization capability of the proposed CM-YOLO model across various crops and multiple disease conditions, we conducted experiments on extended datasets. The tomato leaf dataset includes four disease categories, while the cotton leaf dataset incorporates two additional disease states—early blight and wilt—to cover a wider range of leaf conditions and disease manifestations. Table 8 presents the performance of CM-YOLO compared with the baseline YOLOv8n on key metrics for both the tomato and cotton extended datasets.

**Table 8:** Generalization Experiments.

Method	Tomato				Cotton Leaf			
	P	R	mAP50	mAP50-95	P	R	mAP50	mAP50-95
YOLOv8n	0.587	0.825	0.765	0.647	0.903	0.916	0.939	0.888
CM-YOLO	0.666	0.868	0.790	0.648	0.929	0.921	0.941	0.891

In the tomato leaf disease generalization experiments, CM-YOLO achieved notable improvements over YOLOv8n in both P and R. Specifically, the mAP50 increased from 0.765 for YOLOv8n to 0.790, while the mAP50-95 remained stable, indicating that CM-YOLO demonstrates stronger overall recognition capability across different disease types and maintains consistent performance across samples of varying difficulty.

In the cotton leaf disease generalization experiments, CM-YOLO outperformed YOLOv8n in both precision and recall. Furthermore, improvements were observed in both mAP50 and mAP50-95, demonstrating that CM-YOLO can maintain stable detection performance across leaves with different states.

Significant variations in leaf morphology, lesion characteristics, and color changes exist across different diseases and plant species, posing challenges for cross-crop and cross-disease detection. The stable performance of CM-YOLO on these diverse datasets indicates that its feature extraction and small-target recognition capabilities can effectively adapt to variations in disease types and plant characteristics, exhibiting strong generalization ability. This not only confirms the model’s excellent performance on experimental datasets but also further supports its potential application in real-world agricultural monitoring scenarios.

### Visual detection effects of CM-YOLO

**Fig. 8:**
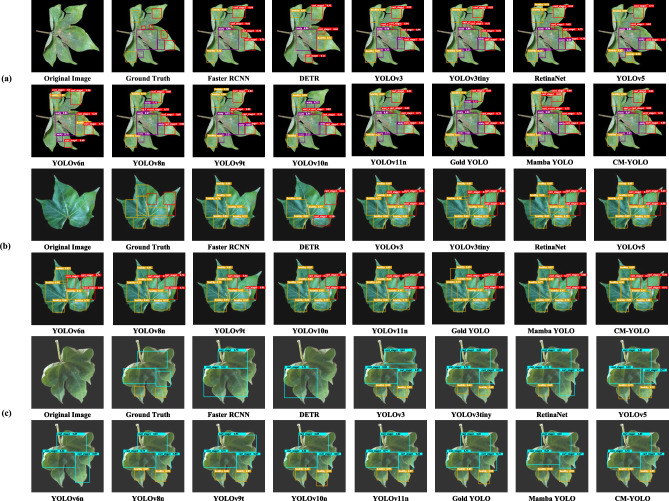
Comparison of model visual inspection results. (a), (b), and (c) correspond to different conditions of cotton leaf diseases.

By incorporating multiple visualizations of detection results into the paper, it becomes possible to intuitively compare the performance of different models under various disease conditions, which helps to comprehensively reveal differences in accuracy, robustness, and the ability to identify subtle lesions^88^. To demonstrate the advantages of CM-YOLO in cotton leaf detection, multiple images were selected from the test set to compare the detection results of different models. The experimental results indicate that CM-YOLO outperforms other models in detecting various states of cotton leaf diseases, with its detection outcomes showing a high degree of consistency with the ground truth in terms of both accuracy and confidence scores. As illustrated in Fig. 8, the figure presents the original images alongside the detection results, allowing for a clear visual observation of the distribution and characteristics of leaf lesions. CM-YOLO provides high-confidence detections and effectively distinguishes overlapping and subtle features, making it the most suitable model for this task. Although YOLOv11n also demonstrates relatively strong performance with high confidence and accurate detection of most disease states, it still exhibits cases with lower confidence, indicating a minor limitation in coverage. In contrast, YOLOv8n and YOLOv10n show moderate performance: while achieving reasonable accuracy, they encounter difficulties in handling overlapping states and maintaining confidence in certain regions. Furthermore, the remaining models perform considerably worse. For example, DETR and Faster R-CNN show significant shortcomings in detecting fine-grained diseases, frequently leading to missed detections and false positives. Their limited ability to address subtle lesions and multi-scale features highlights deficiencies in both precision and robustness.

**Fig. 9:**
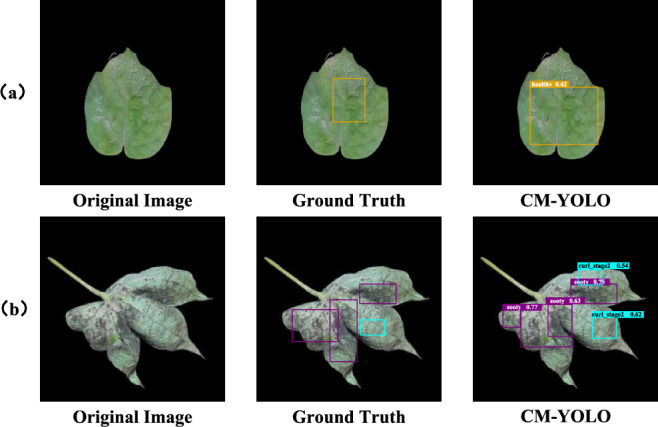
Failure cases. (**a**) and (**b**) correspond to different conditions of cotton leaf diseases.

As shown in Fig. 9, CM-YOLO still exhibits detection errors in certain scenarios. Specifically, Fig. 9(a) illustrates cases where some lesions were incorrectly identified as healthy regions, while Fig. 9(b) demonstrates missed detections of small-scale lesions. This is mainly due to the small lesion size and their similarity in color to healthy leaves, which pose challenges for the model in feature extraction and discrimination. To address this issue, future research could further optimize the feature extraction module by incorporating multi-scale feature fusion and attention mechanisms, thereby improving the recognition of tiny lesions and enhancing the model’s robustness and coverage in complex scenarios.

## Conclusion

This study proposes CM-YOLO, a cotton leaf detection model based on an improved YOLOv8 framework, aimed at enhancing the scientific management and efficiency of cotton cultivation. First, the integration of the SS2D module into the backbone network improves the efficiency and comprehensiveness of feature extraction, enabling the model to capture fine-grained details of small, subtle, and overlapping disease lesions in cotton leaves. Second, the MSDA attention mechanism guides the model to focus on key regions while reducing redundant feature interference, thereby enhancing detection accuracy and robustness. Moreover, the DIoU loss function optimizes anchor box center matching, accelerating model convergence and improving localization precision. Experimental results demonstrate that CM-YOLO outperforms other comparative models in cotton leaf detection, achieving a precision of 0.902 and an mAP50 of 0.933. Simultaneously, the model maintains high detection accuracy while effectively controlling parameter size and computational cost, demonstrating a favorable balance between accuracy and efficiency. Additionally, ablation studies conducted across multiple plant datasets show that CM-YOLO consistently achieves high precision and recall, further validating its stability and applicability in handling diverse disease features and various scenarios. Overall, CM-YOLO not only excels in cotton leaf disease detection but also exhibits strong cross-crop generalization ability, providing a reliable technical foundation for the future implementation of automated and precision agriculture.

Nevertheless, CM-YOLO has certain limitations. First, the model may still miss or assign low-confidence scores to lesions with colors highly similar to the healthy leaf regions. Second, robustness under extreme weather conditions, strong illumination, or other complex environments remains to be further improved. In addition, although CM-YOLO demonstrates good generalization across multiple plant datasets, significant differences in leaf morphology, texture, and disease characteristics across crops require further optimization and fine-tuning when transferring the model to entirely new crops or environments. Therefore, future research may focus on the following directions: (1) incorporating more refined attention mechanisms to further enhance the extraction of micro-lesion features; (2) integrating multimodal information, such as leaf spectral data and environmental parameters, to improve model robustness under complex conditions; (3) exploring model compression and acceleration techniques to enable real-time detection with lower computational overhead, supporting deployment on edge devices and intelligent agricultural hardware; (4) expanding cross-crop and cross-region datasets to further validate and enhance the model’s generalization capability and practical applicability.

## Data Availability

The datasets generated during and analysed during the current study are available from the corresponding author on reasonable request.
